# Reliability of marginal bone level measurements on digital panoramic and digital intraoral radiographs

**DOI:** 10.1007/s11282-019-00387-0

**Published:** 2019-04-19

**Authors:** Kristina Hellén-Halme, Agneta Lith, Xie-Qi Shi

**Affiliations:** 1 Department of Oral and Maxillofacial Radiology, Faculty of Odontology, Malmö University, 205 06 Malmö, Sweden; 2 Department of Oral and Maxillofacial Radiology, Institute of Odontology, The Sahlgrenska Academy, University of Gothenburg, Gothenburg, Sweden; 3 Section of Oral and Maxillofacial Radiology, Department of Clinical Odontology, Faculty of Medicine, University of Bergen, Bergen, Norway

**Keywords:** Marginal bone level, Digital radiology, Panoramic image, Intraoral image, Observer variations

## Abstract

**Objectives:**

The aim of this study was to evaluate the reliability of bitewing and panoramic radiographs in marginal bone level measurements in terms of inter- and intra-observer agreement.

**Methods:**

Fifty paired bitewing and panoramic images were used. Eight observers measured marginal bone level at the mesial and distal surfaces of tooth 25 and tooth 35. Thus, in total 100 radiographs of 400 measurements were obtained for each observer. To evaluate intra-observer agreement, three observers re-evaluated the radiographs after a minimum of 1 month. Intra-class correlation coefficient (ICC) was applied to evaluate the inter- and intra-observer agreement. The *t* test was applied to assess possible difference in measurement between bitewing and panoramic radiographs.

**Results:**

The mean ICC value of inter-observer agreement was 0.85 for bitewing and 0.66 for panoramic radiographs. The mean intra-observer agreement was 0.92 and 0.76 for bitewing and panoramic radiographs, respectively. There was no statistically significant difference between bitewing and panoramic radiographs in measurements of marginal bone level on maxillary tooth 25, whereas a statistically significant difference was found between the two image modalities on mandible tooth 35.

**Conclusion:**

Bitewing examination should be the choice of image modality for assessment of marginal bone level at premolar region due to good to excellent reliability and low radiation dose. However if a panoramic radiograph already exists, a rough estimation of marginal bone level at premolar region is clinically acceptable bearing in mind that the bone height of the mandible premolar region might be overestimated as compared to bitewing radiograph.

## Clinical relevance

*Scientific rationale for the study* It is important to evaluate the reliability of the most commonly used two radiographic methods on marginal bone level assessment. Our findings will assist clinicians in the choice of imaging modality and in the correct interpretation of radiographic examinations on marginal bone height using bitewing and panoramic radiographs.

*Principal findings* This study confirmed previous studies using analogue radiograph that bitewing was more reliable than panoramic radiography in terms of observer agreement. However, the observer agreement applying panoramic imaging was at least moderate and no statistical significant difference was found in distance measurement on maxillary premolar region, indicating when an approximate evaluation of marginal bone level is desired panoramic images may be used, taking into consideration bone height maybe slightly overestimated in the mandible premolar region.

## Introduction

Intraoral and panoramic radiography are the most commonly used image modalities in a dental clinic for marginal bone level assessment. Several studies have compared analogue panoramic and intraoral radiographic images with respect to image quality and accuracy of marginal bone level measurements.[1–5]. Ivanauskaité et al. [4] showed that the diagnostic information obtained from a panoramic radiograph is valuable and useful for evaluating teeth and hard tissues. In a later study, the image quality of bitewing and digital panoramic radiographs in terms of assessment of marginal bone was investigated; the authors concluded that digital panoramic radiograph was sufficient as compared to bitewing radiograph for evaluating marginal bone tissue in the mandibular but not in the maxillary premolar and molar regions [5]. Another study [1] found that panoramic radiographs were comparable with intraoral X-ray regarding the diagnostics of the marginal bone level, whereas a study from Pepelassi et al. [3] found that the panoramic radiograph had a lower diagnostic value. Panoramic radiographs were found to be as reliable as conventional intraoral radiographs when used to assess the point of bone attachment to implant threads [6].

The quality of today’s digital panoramic radiographs has now surpassed that of analogue radiographs, due to technical improvements such as laser alignment lights, more accurate movement patterns adjustable according to patient size and jaw form, more consistent focus layer and digital measuring tools. It is therefore clinically relevant to update the knowledge of digital panoramic radiograph on marginal bone level assessment.

For assessing and monitoring marginal bone level, clinical and radiographic examinations using bitewing and panoramic radiographs are most commonly used in the clinic. One study [7] demonstrated that compared to a full mouth intraoral radiographic examination, a panoramic image supplemented with selected intraoral radiographs reduces the number of exposures. If today´s panoramic radiographs are diagnostically valuable in marginal bone level assessments for entire or part of the jaw bones, the supplementary intraoral radiographs may be further reduced leading to reduced radiation dose and higher patient compliance. To our knowledge, no previous studies have evaluated observer differences in marginal bone level measurements between digital bitewing and panoramic radiographs. Thus, the aim of this study was first to investigate the reliability of digital panoramic and intraoral radiographs in terms of observer agreement on marginal bone level measurements Second, the validity of panoramic radiography on measurement of marginal bone height was evaluated using bitewing as the reference method. The hypotheses were that the reliability of both imaging techniques was comparable in terms of observer agreement, and marginal bone height obtained from panoramic radiograph was comparable to that of bitewing radiograph.

## Material and methods

### Digital radiographs

With 95% confidence level, binomial distribution and a margin of error 0.1, this study would need at least 48 patients if 30% of the diagnosis would vary according to power analysis. 50 cases that had both panoramic and bitewing radiographs were retrospectively selected by a radiographer working at the Faculty, who was otherwise not in the project, from an imaging database at the Faculty of Odontology, Malmö University. Cases were selected consecutively from January 2014 until the number of 50 was reached. The cases were from patients coming to the Faculty for check-ups, usually once in every 2 years. No one had a specific need for specialist care in periodontology. A total number of 100 Digital Imaging and Communications in Medicine (DICOM) [8] images comprised of 50 pairs of panoramic and bitewing radiographs were included. All the images were anonymized randomly coded from 1 to 100. The only parameter not anonymized was which panoramic image belonged to which bitewing image. Patient consent was obtained, as a routine, at registration for each patient at the Faculty of Odontology, i.e., anonymized data in the journal may be used for research and educational purposes.

The case selection criteria were as follows:

1. The panoramic and bitewing images for each case were exposed at maximum 3 months apart.
2. All images depicted the mesial and distal sites on tooth 25 and 35, the sites chosen for marginal bone level measurements.
3. All the panoramic radiographs were acquired with a Veraviewepocs 3D unit [Morita, Kyoto, Japan] and the bitewing images were exposed with a Prostyle Intra X-ray unit and a CMOS ProSensor intraoral sensor/Planmeca Oy, Helsinki, Finland).

The images were presented using the Synedra View software (Synedra IT GmbH, Innsbruck, Austria). Assessments were performed in a room with dimmed ambient light (illuminance less than 50 lx). All monitors applied were of the same type: Olorin^®^ Vista Line VL191D BARTEN (Billdal, Sweden), and were calibrated with a pre-calibration curve per Barten [9].

### Observers

Eight senior students in their final year of dental education at the Faculty of Odontology dental school, volunteered to be observers. All had gone through their education in radiology and passed the final exam in this subject.

The marginal bone levels at the mesial and distal sites on teeth 25 and 35 were measured by all the observers. Measurements were made from the cemento-enamel junction (CEJ) to the marginal bone level on both the panoramic and bitewing images and noted on the protocol, using the measuring tool in Synedra View. The definition of marginal bone level, was set to be the highest point of bone coverage at each site. If two bone levels were seen in the radiographs, the measurement was made of the lowest one in both modalities. If uncertainty of measurement occurred, comments could be registered for each case by each observer. All measurements on the panoramic and the intraoral images were corrected for the magnification. Observers were allowed to zoom in and out and adjust the brightness and contrast of the radiographs according to their own preference.

To establish intra-observer agreement, three of the eight observers were randomly selected to repeat the same assessment at least 1 month later.

### Statistics

All measurement data were analyzed using the Statistical Package for the Social Sciences (SPSS; IBM New York, USA). An analysis of all measurements was performed to reveal any outliers. Both inter-observer agreement and intra-observer agreement were presented using the intra-class correlation coefficient (ICC. 2.1). Intra-observer agreement calculation was based on three observers’ data. Paired *t* test was applied to assess possible difference in measurements between bitewing and panoramic radiographs.

## Results

One apparent outlier was found and was considered as a clerical error, thus excluded. Table 1 presents inter-observer agreement for the eight observers. The average ICC for the bitewing and panoramic radiographs was 0.85 and 0.66, respectively. For the bitewing images, the highest inter-observer agreement (0.95) occurred in measurements at tooth 25, the distal site, and lowest agreement 0.64 at tooth 35, the mesial site. According to Koo and Li [10], this indicates an inter-observer agreement between moderate to excellent for measurements in bitewing images.

**Table 1:** Inter-observer agreement for eight observers assessing marginal bone level on digital bitewing and panoramic radiographs at four sites (25m = tooth 25, mesial site; 25d = tooth 25, distal site; 35m = tooth 35, mesial site; 35d = tooth 35, distal site)

	Inter-observer agreement					
	Bitewing images			Panoramic images		
	ICC	95% confidence interval		ICC	95% confidence interval	
		Lower end	Upper end		Lower end	Upper end
25m	0.87	0.80	0.92	0.64	0.54	0.75
25d	0.91	0.86	0.95	0.67	0.56	0.77
35m	0.77	0.64	0.86	0.63	0.50	0.75
35d	0.86	0.79	0.92	0.71	0.58	0.82

*ICC* Intra-class correlation coefficient

For the panoramic images, the highest inter-observer agreement (0.82) was found in measurement at tooth 35, distally, and the lowest agreement (0.50) for tooth 35, mesially. This indicates an inter-observer agreement between moderate to good for measurements in the panoramic images [10].

Table 2 presents the ICCs and 95% CIs for intra-observer agreement for three observers who assessed the images twice. The mean of all measurement points in bitewing images was 0.92 and in panoramic images, 0.76. The highest agreement between the measurements of an observer was 0.97 on a bitewing X-ray image for tooth number 25 distally; the lowest intra-observer agreement was 0.44 on a panoramic image for tooth number 35 mesially.

**Table 2:** Intra-observer agreement for three observers (A, B, and C) assessing marginal bone level on digital bitewing and panoramic radiographs at four sites (25m = tooth 25, mesial site; 25d = tooth 25, distal site; 35m = tooth 35, mesial site; 35d = tooth 35, distal site)

	Intra-observer agreement					
	Bitewing images			Panoramic images		
	ICC	LE	UE	ICC	LE	UE
A						
25m	0.95	0.90	0.97	0.67	0.48	0.80
25d	0.95	0.91	0.97	0.75	0.60	0.85
35m	0.90	0.81	0.95	0.91	0.85	0.95
35d	0.95	0.90	0.98	0.91	0.84	0.95
B						
25m	0.91	0.80	0.95	0.88	0.78	0.93
25d	0.94	0.85	0.97	0.86	0.76	0.92
35m	0.82	0.39	0.93	0.44	0.44	0.65
35d	0.90	0.79	0.95	0.82	0.64	0.91
C						
25m	0.92	0.86	0.95	0.70	0.53	0.82
25d	0.97	0.95	0.98	0.73	0.57	0.84
35m	0.88	0.74	0.94	0.63	0.39	0.79
35d	0.94	0.88	0.97	0.79	0.63	0.88

*ICC* Intra-class correlation coefficient, *LE* lower end of the 95% confidence interval (CI), *UE* upper end of the 95% CI

**Fig. 1:**
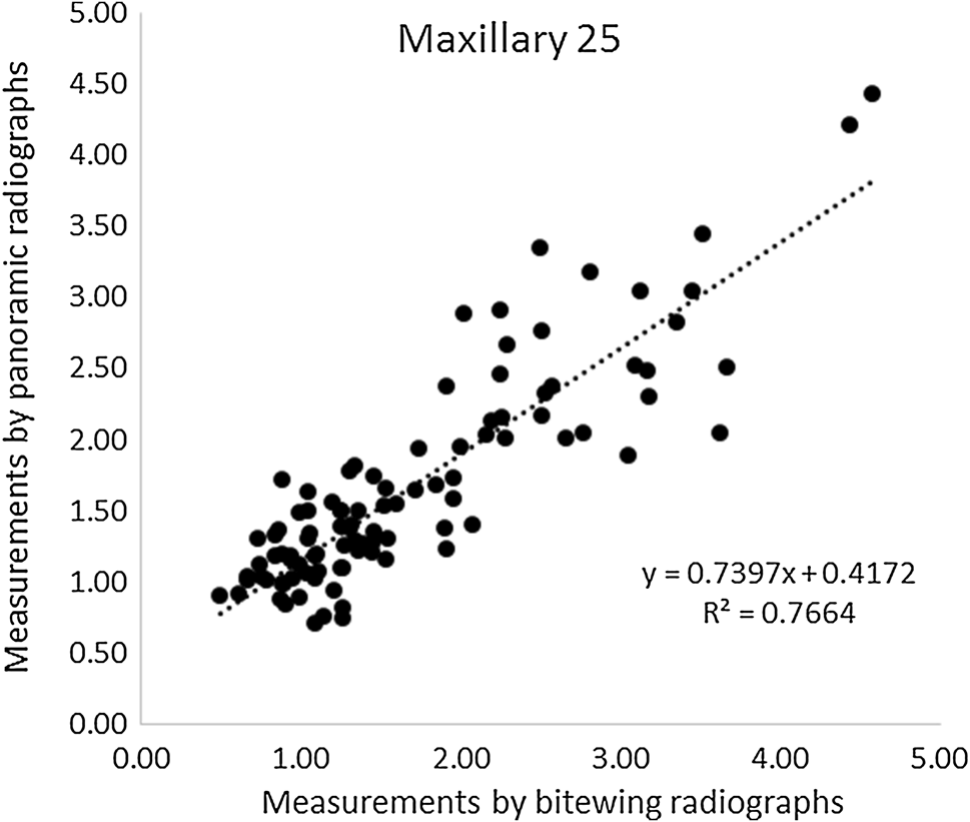
Illustration of the linear correlations between measurements performed using bitewing and panoramic radiographs for tooth 25

**Fig. 2:**
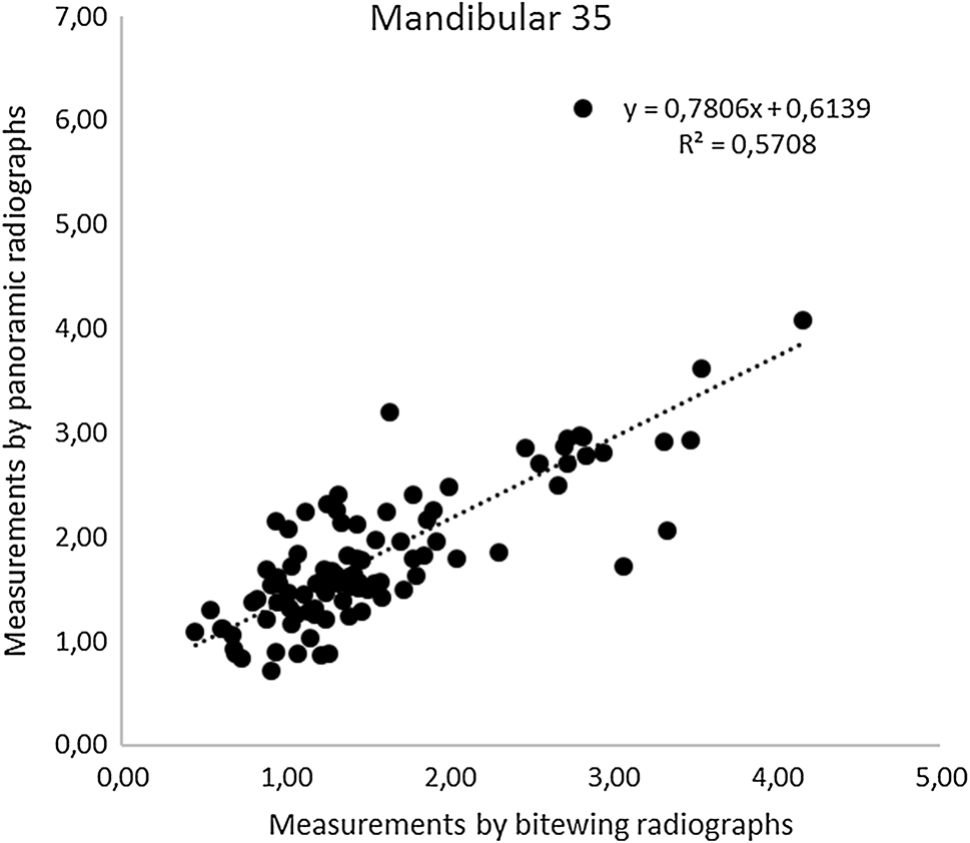
Illustration of the linear correlations between measurements performed using bitewing and panoramic radiographs for tooth 35

Figures 1 and 2 illustrate linear correlations between measurements performed using bitewing and panoramic radiographs for tooth 25 and tooth 35, respectively. The correlation coefficient was 0.77 for measurements performed on tooth 25 and 0.57 for tooth 35. Paired *t *test showed no statistically significant difference between bitewing and panoramic radiographs in measurements of marginal bone level on maxillary tooth 25 (*p* = 0.59) with a mean difference of 0.02 mm, whereas a statistically significant difference was found between the two image modalities on mandible tooth 35 (2.01718E−06) with a mean difference of − 0.27 mm, indicating that measurements on panoramic radiographs tended to result in a higher value.

The observers noted 39% of the panoramic images as uncertain assessments, and the corresponding uncertain measurements was 5% for bitewing images.

## Discussion

The present study investigated how marginal bone level measurements of the second premolar in the upper and lower jaws differed between digital bitewing and panoramic radiographs. The chosen areas were related to the fact that the panoramic images in this particular sites usually show overlapping of teeth and marginal bone level, making it more difficult on diagnostics of caries and marginal bone levels. Our results found that measurements done on bitewing images were more consistent between and among observers than that on panoramic images. The consistency was, as expected, higher for intra-observer agreement than inter-observer agreement. Overall, the ICC was lower for measurements made on panoramic images, meaning that when only panoramic images are available clinically, marginal bone level measurements will differ to a higher extent than they would have if measured on bitewing images. Nevertheless, the reliability of the panoramic radiograph was within the range of moderate to good, and thus might be considered as clinically acceptable.

Difference in marginal bone level between the two image modalities was statistically significant in the mandibular premolar region. This finding is contradictory to some earlier studies using the analogue radiographic technique, in which they have shown that clinicians underestimated marginal bone loss in panoramic images [1, 4]. This discrepancy between the studies may be due to differences in digital and analogue technique where the measurement methods differ. Digital technology eliminates potential sources of analogue measurement error, where radiographic film is scanned, calibrated, and then magnified and measured with a ruler or slider. The distortion of vertical measurements on panoramic imaging results from the fact that the radiation source is normally 5°–10° upward from the lingual side. The inclinations of alveolar processes in maxillary and mandible at different region may also play a role. The mean difference in bone height was 0.27 mm in region of 35, whether this small difference had clinical significance that needs to be interpreted with caution.

The validity of both methods on assessment of marginal bone level could not be studied, since this is a retrospective clinical study and thus no “gold standard” could be achieved. Using bitewing as the reference method, we found no statistically significant difference on marginal bone measurement in the upper premolar region. To what extent patient treatment varies when applying these two radiographic methods have not been evaluated in this study. Periodontal diagnoses are based on various types of measurements, and choice of treatment is not decided on radiographic results alone. A comprehensive assessment of clinical and radiological appearance governs treatment choice.

The purpose of this study was to evaluate the method itself rather than observer ability. The number of observers can affect the evaluation of a method, thus several observers are required in studies such as this. Studies have found that more than six observers does not increase accuracy when assessing a method [11, 12]. Caries diagnostic studies have shown that the strength of the statistical calculation increases with the number of observers times the number of assessed areas, up to a certain limit. The number of observers in relation to the number of areas is inconsequential, as long as the total number of observations per method is the same [12, 13].

In bitewing images of high quality, the marginal bone level could be clearly distinguished, and all observers measured the bone level with a high degree of consistency. The observer consistency was somehow lower when panoramic images were applied. The observers experienced uncertainty marginal bone level measurement in 39% of the panoramic image, whereas the number was only 5%. This was expected since panoramic radiography has poorer resolution and more proximal overlaps, causing larger differences in marginal bone level measurements compared with bitewing images.

The observers were dental students in their final year with limited clinical experience in evaluating radiographic images, however, identifying ECJ and marginal bone level was considered more as pattern recognition than diagnostics. Intra- and inter-observer agreement of experienced general practicing dentists or specialists in oral and maxillofacial radiology might have been even better.

## Conclusion

Bitewing examination should be the choice of image modality for assessment of marginal bone level at premolar region due to good to excellent reliability and low radiation dose. However if a panoramic radiograph already exists, a rough estimation of marginal bone level at premolar region is clinically acceptable bearing in mind that bone height of the mandible premolar region might be overestimated as compared to bitewing radiograph.
